# Correction: Inhibition of cell movement and proliferation by cell–cell contact-induced interaction of Necl-5 with nectin-3

**DOI:** 10.1083/jcb.20050109005122020c

**Published:** 2020-05-15

**Authors:** Tsutomu Fujito, Wataru Ikeda, Shigeki Kakunaga, Yukiko Minami, Mihoko Kajita, Yasuhisa Sakamoto, Morito Monden, Yoshimi Takai

Vol. 171, No. 1 10.1083/jcb.200501090 October 10, 2005.

An error was noticed in the “siRNA experiments” section of the Materials and methods of this paper. The sequence provided for the nectin-3 siRNA (5′-UGAUCAAUGUGCUGUUCAA-3′) is incorrect. The correct sequence is 5′-GGACAUUCGCUACUCUUUCAUACUA-3′.

